# Pathological intracranial extradural hematoma in a 10-year-old child

**DOI:** 10.4103/1817-1745.76121

**Published:** 2010

**Authors:** Abdul Rashid Bhat, Ashish Kumar Jain, A. R. Kirmani, Furqan Nizami

**Affiliations:** Department of Neurosurgery, Sher-i-Kashmir Institute of Medical Sciences (SKIMS), Srinagar, Kashmir, India

**Keywords:** Calvarium, eosinophilic granuloma, extradural hematoma

## Abstract

A nontraumatic spontaneous extradural hematoma, in a fully conscious 10-year-old male child, caused by a solitary eosinophilic granuloma of calvarium presented as a case of localized painful swelling of the head, which rapidly expanded and decreased in size. A plain CT-scan of the head with bone window revealed eroded right parietal bone with subperiosteal debris and extradural hematoma of mixed density. Immediate evacuation of the extradural clot and complete excision of the lesion was performed to prevent the deterioration of the patient and to achieve the histological diagnosis for further management.

## Introduction

Extradural hematomas of nontraumatic and pathological origin have been reported occasionally for the last six decades.[1,2] There have been many pathological causes of this fatal complication from sinus infection to coagulopathies and primary tumors to metastasis of skull and dura.[2–8] But still this entity is uncommon. We report an extradural hematoma in a 10-year-old boy as a complication of solitary calvarial eosinophilic granuloma.

## Case Report

**Figure 1:**
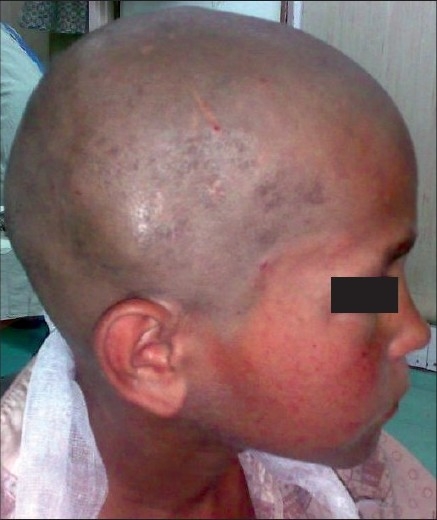
Non-traumatic, diffuse, tender and pulsatile right parietal swelling.

**Figure 2:**
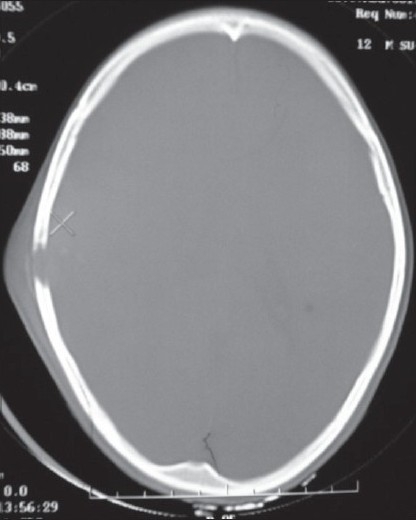
Plain CT-scan of head (bone window) showing lytic area in the right side of the parietal calvarium.

**Figure 3:**
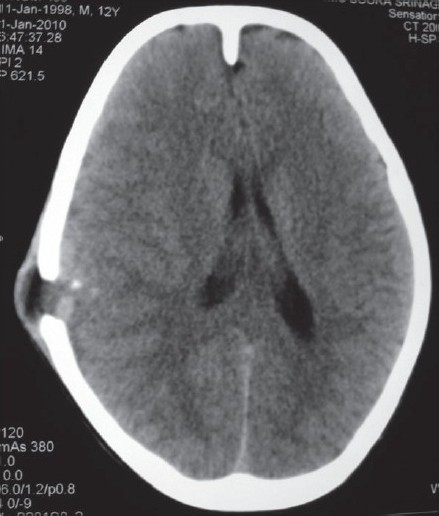
Plain CT-scan of head revealing erosion in the parietal bone with subperiosteal debris.

**Figure 4:**
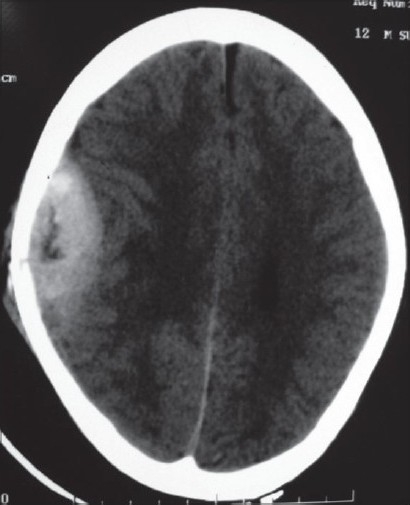
NCCT brain showing right parietal extradural hematoma with fresh hemorrhage under lytic inner table of skull.

**Figure 5:**
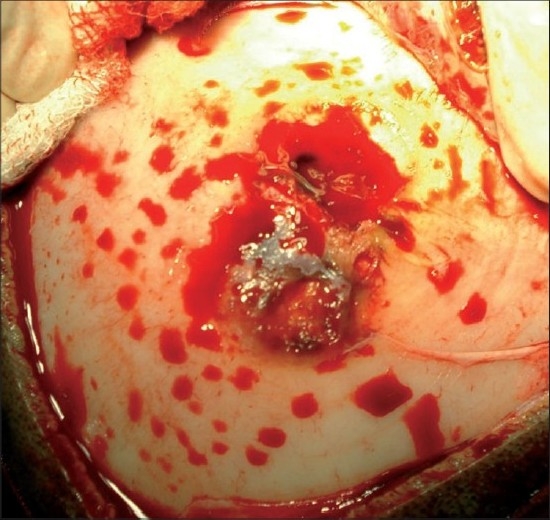
Intra-operative photograph showing eroded bone with the lesion.

**Figure 6:**
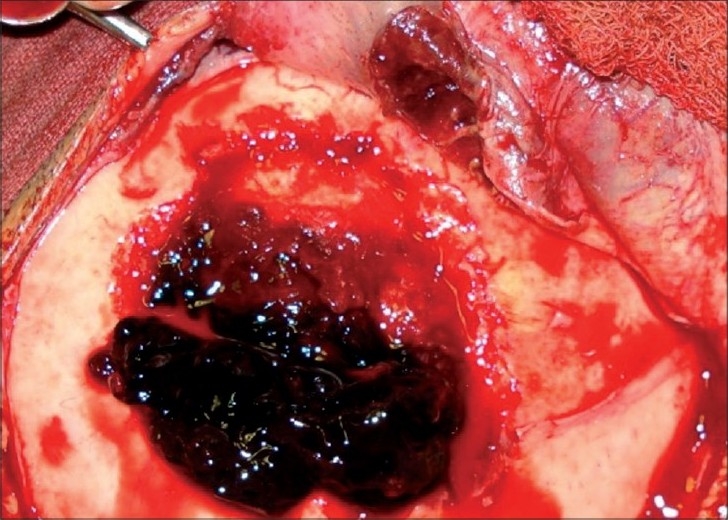
Extradural hematoma seen after excision of lesion and perilesional bone.

**Figure 7:**
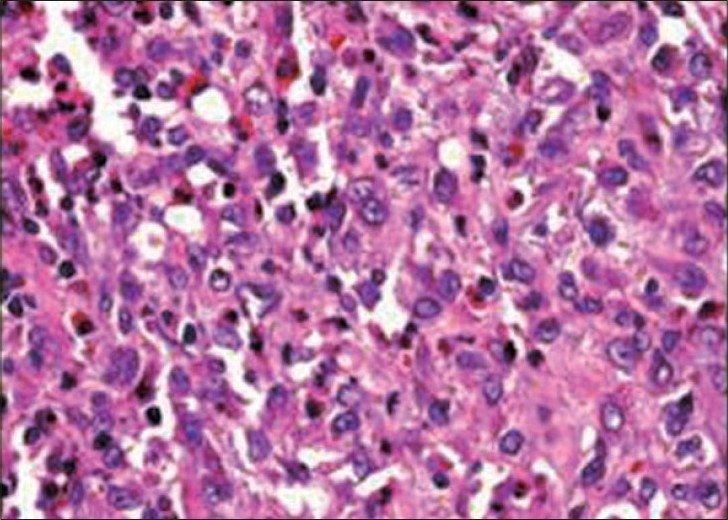
Showing eosinophils and Langerhans cells, (H&E, ×25)

A 10-year-old, fully conscious, boy presented to the accident and emergency room of Neurosurgical Centre, Sher -i- Kashmir Institute of Medical Sciences (SKIMS), Kashmir, with one month history of a gradually appearing almond-sized, swelling on the right side of the head with dull aching pain. The swelling suddenly increased to the size of a walnut with increase in the intensity of pain for last 15 days. However, for the last one week, the swelling rapidly decreased in size but became pulsatile and at the same time pain continued unabated. There was no history of trauma, episodes of unconsciousness, headache, vomiting, drug intake, fever, hemoptysis, hematuria or abdominal pain. Clinically, the patient was fully conscious and intelligent too. General physical examination was normal. Locally a single, 3 × 3 × 4 cm, diffuse, pulsatile, soft and compressible but tender swelling was found on the right parietal region [Figure 1]. The temperature of the swelling was slightly raised than rest of the body. The basic routine blood and radiological investigations were performed. A plain CT-scan of brain with bone window showed a lytic lesion of 3 × 3 cm in the right parietal bone [Figure 2] with overlying subperiosteal debris [Figure 3] and an underlying mixed density extradural hematoma (EDH) of about 20 ml [Figure 4]. The patient was prepared for surgery to prevent further morbidity. The periosteum was studded and adherent to a pinkish, vascular soft mass. The local skull had a defect of 3 × 4 cm with beveled margin. The extradural hematoma was seen under the defect. The subperiosteal debris and the eroded bone were excised completely after curettage and the EDH evacuated [Figures 5 and 6]. The post operative period was uneventful. The histopathological examination of the debris, lytic bone and clots revealed eosinophilic granuloma with both eosinophils and Langerhans cells and the lesion infiltrating up to dura [Figure 7].

## Discussion

The spontaneous, nontraumatic or pathological extradural hematomas are uncommon. Their presentation depends upon the nature of underlying lesion, pain and tenderness locally, swelling of the scalp or rise in the intracranial pressure. The eosinophilic granulomas (a type of histiocytosis X) are solitary, nonrecurring and moderately vascular lesions of the skull found in older children and young adults. Local tenderness and pain usually call attention to the lesion. It is rare for these lesions to bleed extradurally.[9–11] At SKIMS, Kashmir, it was rare for the age (10 years) of our patient to have eosinophilic granuloma and moreover rare for such a lesion to bleed into extradural space. A study reviewed that there have been only 19 cases of spontaneous EDH reported in the literature till June, 2009.[2] Most of the cases have been due to paranasal infections[5,12–15] and chronic otitis media[1,2,16–18] The radiation therapy to the pleomorphic sarcoma like malignant fibrous histiocytoma of dural meninges has been incriminated as a cause of spontaneous EDH.[4] The metastatic lesions from the hepatocellular carcinoma and lung cancer to the skull have been reported to bleed and cause extradural hematoma.[6,8] However, these lesions are very vascular and vulnerable to bleeding. The proposed mechanism of extradural hemorrhage (EDH) in paranasal infections and otitis media was infection leading to the vasculitis and thereby rupture of vasculitic vessels and subsequent expansion[3,14,19,20] Although intradiploic epidermoids are believed to cause EDH by erosion of the intradiploic and skull vessels,[7] the possible causes of the EDH of our case at SKIMS, Kashmir are stripping of dura from bone due to rapid tumor growth, expansion (bleeding in tumor), erosion of the intradiploic vessels, erosion of the normal dural surface vessels and bleeding of localized dural neovascularization caused by the eosinophilic granuloma. We suggest prompt CT-scan of the head in case of pain and tenderness in an existing head swelling or a rapidly expanding and regressing swelling in size with pain. But clinical condition of the patient is the most accurate indication of an investigation, since such a case can turn up as extradural hematoma may not hold true every time.
